# The use of light, temperature and pressure in the treatment of depression

**DOI:** 10.1192/j.eurpsy.2021.1961

**Published:** 2021-08-13

**Authors:** D. Zalewski, A. Nobis, E. Dąbrowska, N. Waszkiewicz

**Affiliations:** Department Of Psychiatry, Medical University of Białystok, Choroszcz, Poland

**Keywords:** light, Depression, temperature, pressure

## Abstract

**Introduction:**

Climate and weather have a great influence on the prevalence of depressive disorders. Selected physical parameters for instance light, temperature and pressure can be used to treat mood disorders.

**Objectives:**

The present mini-review aims at approximating the mechanisms by which selected, strictly controlled physical parameters in particular light, temperature, and oxygen pressure can help in the treatment of depression and determine their potential effectiveness.

**Methods:**

Relevant literature was identified by searching the PubMed/Medline database, by combining the search strategy of free text terms and exploding a range of MESH headings relating to the topics.

**Results:**

Mechanisms that can modify the course of depression were briefly presented. Review of the literature showed the well-established position of bright light therapy (BLT) effective in treating seasonal (SAD) and non-seasonal affective disorders (non-SAD); safety and rapid-action of whole-body hyperthermia (WBH) and whole-body cryotherapy (WBC) were also demonstrated; the least data was available on hyperbaric oxygen therapy (HBOT), which had antidepressant properties only in people with concomitant neurological damages.

**Conclusions:**

In addition to the well-established position of BLT in the treatment of depression, further research is needed on other methods, such as WBH, WBC, HBOT.

**Disclosure:**

No significant relationships.

